# Expanding the ESMO-MCBS: integrating organ preservation into clinical benefit assessment in rectal cancer

**DOI:** 10.1016/j.esmogo.2026.100417

**Published:** 2026-09-21

**Authors:** E.S. Bergen, G. Piringer, F. Aigner, F. Roeder, G.W. Prager, L. Weiss

**Affiliations:** 1 Division of Oncology, Department of Medicine 1, Medical University of Vienna, Vienna, Austria; 2 Austrian Breast & Colorectal Cancer Study Group (ABCSG), Vienna, Austria; 3 Department of Hematology and Oncology, Kepler University Hospital and Johannes Kepler University Linz, Linz, Austria; 4 Department of Surgery, Barmherzige Brüder Krankenhaus Graz, Graz, Austria; 5 Department of Radiotherapy and Radiation Oncology, Paracelsus Medical University, Salzburg, Austria; 6 Department of Internal Medicine III, Paracelsus Medical University, Salzburg, Austria

We are writing to address the current application of the ESMO Magnitude of Clinical Benefit Scale (MCBS) in the specific context of rectal cancer. The MCBS is a widely adopted framework for assessing clinical benefit in oncology that has improved the comparability across trials by focusing on robust study endpoints, such as disease-free (DFS) and overall survival (OS),^1^ but it does not fully capture clinically meaningful benefits specific to rectal cancer—particularly organ preservation. We therefore believe there is an opportunity for further refinement of this scoring system, in light of evolving multimodal treatment strategies in rectal cancer.

Organ preservation, defined as the omission of total mesorectal excision (TME) after neoadjuvant treatment and encompassing watch-and-wait and local excision strategies, has gained increasing importance in patients with distal rectal cancer, as reflected by several phase II and III trials incorporating this endpoint (Table 1). This is highly important from a patient perspective, as radical surgery for distal rectal cancer is frequently associated with substantial impairment of quality of life, including permanent colostomy or a high risk of low anterior resection syndrome.

**Table 1:** Selection of phase II and III studies in rectal cancer including organ preservation endpoints

Trial	Design	Population	Intervention	NOM-based endpoint	Reference
OPRA	Phase II, randomized	Stage II-III rectal cancer	Induction versus consolidation CHT + CRT, selective NOM	TME-free survival	Garcia-Aguilar et al.^2^
GRECCAR 2	Phase III, randomized	cT2-3 low rectal cancer with good response after CRT	LE versus TME after CRT	Composite outcome (death, recurrence, morbidity, side-effects) at 2 years	Rullier et al.^3^
ACOSOG Z6041	Phase II, single-arm	cT2N0 distal rectal cancer	Neoadjuvant CRT + LE	Proportion avoiding TME	Garcia-Aguilar et al.^4^
STAR-TREC	Phase II/III, randomized	Early rectal cancer (cT1-T3a/b)	Short-course RT or CRT ± LE versus TME	Rate of organ preservation at 30 months	Rombouts et al.^5^
MSK Dostarlimab	Phase II, single-arm	dMMR rectal cancer, stage II-III	Dostarlimab	cCR at 12 months	Cercek et al.^6^
OPERA	Phase II, randomized	cT2-T3 low rectal cancer	Contract X-ray brachytherapy + CRT versus CRT alone	3-year organ preservation rate	Gerard et al.^7^

cCR, clinical complete response; CHT, chemotherapy; CRT, chemoradiation; dMMR, deficient mismatch repair; LE, local excision; NOM, nonoperative management; TME, total mesorectal excision.

Although chemoradiation followed by surgery and adjuvant chemotherapy long represented the standard of care, trials such as RAPIDO and PRODIGE-23 established total neoadjuvant therapy (TNT) as a preferred approach in intermediate- and high-risk disease, demonstrating improvements in pathological complete remission, DFS, and OS.^8^

The OPRA trial illustrates a limitation of the current MCBS.^2^^,^^9^ In this randomized phase II trial, the two TNT sequencing strategies achieved comparable DFS (primary endpoint), demonstrating oncological equivalence. However, a statistically and clinically significant improvement in TME-free survival was observed for the treatment arm with chemoradiotherapy followed by chemotherapy. However, because this advantage was observed in a secondary rather than the primary endpoint, the study remains unscorable within the current MCBS framework. As a result, a clinically important TME-free survival benefit is effectively ignored despite the absence of any loss in cancer-related outcomes.

In parallel, TME-free strategies are further supported by highly responsive subgroups. In deficient mismatch repair/microsatellite instable disease, treatment with the immune checkpoint inhibitor dostarlimab yielded a 100% clinical complete remission rate, strongly supporting organ preservation in this distinct molecularly defined subgroup.^6^ These findings shift attention toward low- and intermediate-risk distal disease, where local excision with or without radiotherapy and watch-and-wait strategies have demonstrated outcomes comparable to TME, often requiring intersphincteric resection or sphincter excision.^10^

Traditional endpoints such as DFS and OS alone may therefore no longer sufficiently discriminate between treatment strategies, particularly when survival differences are marginal but functional outcomes differ substantially. We therefore propose to incorporate TME-free survival as a distinct dimension of clinical benefit in the MCBS for rectal cancer. Such an extension would complement, not replace, survival endpoints and maintain the scale’s general applicability.

We believe that adapting the MCBS to better capture patient-centered outcomes would substantially improve its relevance in rectal cancer and enable a more meaningful assessment and comparison of emerging treatment strategies that prioritize TME-free survival.
